# Thermal Decomposition of Ternary Sodium Graphite Intercalation Compounds

**DOI:** 10.1002/chem.202000422

**Published:** 2020-04-07

**Authors:** Heather Au, Noelia Rubio, David J. Buckley, Cecilia Mattevi, Milo S. P. Shaffer

**Affiliations:** ^1^ Department of Chemical Engineering Imperial College London London SW7 2AZ UK; ^2^ Department of Chemistry and Materials Imperial College London London SW7 2AZ UK; ^3^ National Physical Laboratory Teddington TW11 0LW UK; ^4^ Department of Materials Imperial College London London SW7 2AZ UK

**Keywords:** 2D materials, exfoliation, graphene, graphite intercalation compounds, X-ray diffraction

## Abstract

Graphite intercalation compounds (GICs) are often used to produce exfoliated or functionalised graphene related materials (GRMs) in a specific solvent. This study explores the formation of the Na‐tetrahydrofuran (THF)‐GIC and a new ternary system based on dimethylacetamide (DMAc). Detailed comparisons of in situ temperature dependent XRD with TGA‐MS and Raman measurements reveal a series of dynamic transformations during heating. Surprisingly, the bulk of the intercalation compound is stable under ambient conditions, trapped between the graphene sheets. The heating process drives a reorganisation of the solvent and Na molecules, then an evaporation of the solvent; however, the solvent loss is arrested by restacking of the graphene layers, leading to trapped solvent bubbles. Eventually, the bubbles rupture, releasing the remaining solvent and creating expanded graphite. These trapped dopants may provide useful property enhancements, but also potentially confound measurements of grafting efficiency in liquid‐phase covalent functionalization experiments on 2D materials.

## Graphical abstract

**Producing graphene**: Graphite intercalation compounds (GICs) are often used to produce exfoliated or functionalised graphene related materials (GRMs) in a specific solvent. This study explores the formation of the Na‐tetrahydrofuran (THF)‐GIC and a new ternary system based on dimethylacetamide (DMAc).

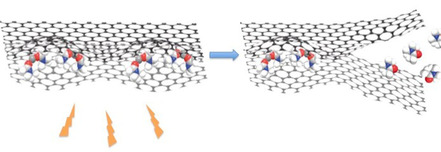

## Introduction

Reductive dissolution of nanocarbons is a versatile and widely used methodology to achieve individualisation, exfoliation and functionalisation of carbon nanotubes (CNTs), fullerenes, and other graphitic nanomaterials;^1^ it has been extended, more recently, to graphene analogues, such as carbon nitride^2^ and transition‐metal dichalcogenides.^3^ For the exfoliation of graphite in particular, graphite intercalation compounds (GICs) are a convenient precursor to obtaining “graphenide” solutions in various solvents, such as tetrahydrofuran (THF) and *N*‐methylpyrrolidone (NMP).^4^, ^5^, ^6^ Alkali metal GICs have long been studied,^7^ but with the sudden growth in graphene research since 2004,^8^ there has been renewed interest in GICs as a possible route to obtaining isolated single‐layer graphene, as well as covalently modified graphene derivatives. Intercalation of alkali metals into graphite to produce well‐defined stage compounds has typically been achieved in the vapour phase;^9^, ^10^ for some alkali metals, direct contact with the molten metal under inert atmosphere also results in well‐staged GICs.^11^, ^12^ Lithium and potassium both insert readily into the graphite interlayer, forming compounds LiC_6_ and KC_8_, but pure sodium does not intercalate graphite to any great extent, forming only high stage compounds (NaC_64_).^7^ However, the insertion of sodium into graphite is possible with the addition of a co‐intercalant species: low and well‐defined stage ternary compounds containing sodium can be readily formed, either as an alloy with caesium or potassium,^7^ or more often, in solution phase with aprotic solvent molecules.^13^ In general, alkali metals dissolved in liquid ammonia generate ternary intercalation compounds with ammonia co‐intercalated between the graphene layers;^5^, ^14^, ^15^, ^16^ organic solvent co‐intercalants include THF, 1,2‐dimethoxyethane (DME), alkyl amines, and even crown ethers.^13^, ^17^, ^18^, ^19^, ^20^, ^21^, ^22^, ^23^ Often, a charge transfer agent such as naphthalene or anthracene is added to the reaction to facilitate the intercalation process in organic solvents.^13^, ^24^ Co‐insertion of sodium with a variety of glymes is also possible by electrochemical means^25^, ^26^ showing good reversibility in charging and discharging.^27^ The particular combination of alkali metal and solvent in these systems is important in determining whether a binary or ternary compound will form;^18^, ^28^ the degree of crystallinity in the starting material has also been found to affect the extent of co‐intercalation.^13^, ^18^

Because intercalation increases the graphite interlayer spacing, especially in low stage index compounds, and introduces potentially repulsive Coulombic interactions, GICs are promising starting materials to produce solvated graphene layers. Alkali metal GICs have been dispersed in a variety of solvents, including, for example, potassium GICs introduced in NMP,^29^ THF,^30^ and even water.^31^ Before these solutions are quenched, the negative charges on the graphenide sheets may be exploited for reaction with suitable electrophiles including diazonium salts,^32^ alkyl,^33^ aryl^34^ and polymer^35^ halides, bromine,^36^ vinyl or acrylate monomers such as styrene^37^ or methyl methacrylate,^38^ and proton donors such as ethanol,^39^ resulting in covalent modification of the graphene sheets.

However, truly spontaneous and complete dissolution is non‐trivial, and is strongly dependent on the alkali metal to carbon stoichiometry. Too little charge means that the van der Waals energy between graphene sheets is greater than the electrostatic repulsion necessary for exfoliation, whilst too much charge causes the cations to remain condensed between graphene layers.^40^, ^41^ This balance is further affected by both the graphite starting material and the overall ion concentration in solution, which can control the degree of exfoliation.^24^ Depending on these factors, the products are more often few layer graphenes (FLGs) rather than true single‐layer graphene (SLG). Even after quenching and washing, residual intercalant can remain inside the material,^36^, ^37^, ^38^ which may cause complications in subsequent applications and material characterisation. In covalently functionalised graphenes, this residual solvent can affect the apparent grafting ratios, which are most commonly quantified by thermogravimetric analysis (TGA).^42^ The presence of residual intercalant also implies an incomplete use of charge during the grafting reaction, and a retained doping of the graphene. Given that these GICs appear to be surprisingly stable even after exposure to ambient conditions, and at temperatures far exceeding the solvent boiling point, a better understanding of the deintercalation behaviour of ternary alkali metal compounds is needed to enable optimisation of both the exfoliation and functionalisation processes.

The intercalation of alkali metals into GICs has traditionally been characterised by X‐ray diffraction (XRD),^13^, ^18^, ^19^, ^20^, ^26^, ^28^, ^43^, ^44^ allowing the stage index to be deduced. In situ XRD has been used to observe the well‐defined stage transitions which occur during the vapour phase insertion of potassium into graphite,^10^ whilst operando measurements have been used to study electrochemical intercalation of sodium and diethylene glycol dimethyl ether (DEGDME).^27^ Raman spectroscopy can monitor the level of charge transfer, and therefore intercalation, in graphite, and is a useful complementary technique for characterising GICs,^18^, ^25^ whilst TGA has been used to calculate the amounts of inserted metal and solvent, and can provide information on structural composition.^17^, ^44^ The decomposition of solvent is observed at surprisingly high temperatures as the GICs are heated, but the structural evolution is often complex and not well understood. Most of these studies focus mainly on metal insertion and do not consider de‐intercalation behaviour, with only a few offering insights into reversibility of the process^10^, ^27^ or stability of the product.^26^, ^45^ Since intercalation appears to be strongly dependent on the choice of metal, solvent and host graphite, de‐intercalation behaviour might also be expected to differ between these systems.

In this work, two ternary sodium GICs were synthesised from natural flake graphite (NFG), and their behaviour studied as a function of temperature. Sodium‐THF‐graphite (Na‐THF‐NFG) is often used for graphene functionalisation,^24^, ^36^, ^38^ and although the intercalation behaviour has been extensively studied, removal of intercalants from the structure is less well understood. *N*,*N*‐dimethylacetamide (DMAc) is a good solvent for the reductive dissolution of CNTs^46^ and carbon nitride^2^ and could be potentially useful for obtaining exfoliated graphene; therefore, sodium‐DMAc‐graphite (Na‐DMAc‐NFG) was also synthesised, and characterised for the first time. These ternary Na‐GICs were characterised with XPS and TGA‐MS. The thermal decomposition was monitored with XRD and correlated to TGA‐MS and Raman, establishing the deintercalation behaviour of these ternary compounds. Understanding this phenomenon is crucial to obtaining pure functionalised graphenes and may also be useful for alternative applications such as Na‐ion batteries.

## Results and Discussion

Two different ternary intercalation compounds were prepared from natural flake graphite, selected for its high crystallinity, using solutions of sodium naphthalide in THF or DMAc. Sodium and naphthalene were dissolved in each solvent, resulting in dark green solutions. In each case, the presence of the naphthalide radical anion was confirmed by UV/Vis spectroscopy, with each solvent system presenting the characteristic double peak at about 800 nm (Figure S1, Supporting Information). After charging with these naphthalide solutions, the graphites turned a metallic blue colour, suggesting that successful intercalation occurred.

**Figure 1:**
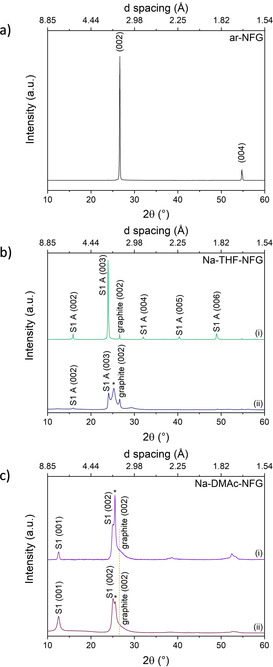
a) XRD pattern of ar‐NFG; XRD patterns of b) Na‐THF‐NFG and c) Na‐DMAc‐NFG (dotted line refers to random stage or turbostratic graphite) before (i) and after (ii) exposure to dry O_2_/N_2_; Cu_K*α*_=1.542 Å. S1A(002), S1A(003), S1A(004), S1A(005) and S1A(006) peaks were assigned according to the peaks described in literature for Na‐THF GICs.^13^

X‐ray diffraction analysis (Cu_K*α*_=1.542 Å) of the two materials confirms the successful formation of ternary graphite intercalation compounds (Figure 1) in each solvent system. In Na‐THF‐NFG, peaks at 15.8°, 23.9°, 32.1°, 40.3°, and 48.9° arise from the (00*l*) planes of a stage one compound with the phase A structure, in which four THF molecules are tetrahedrally coordinated around one sodium ion (Figure S2, Supporting Information).^13^ The interlayer distance for this phase is 11.2 Å, corresponding to an intercalant layer thickness of 7.8 Å.^13^ Significant weakening of the graphite interlayer (002) peak at 2*θ*=26.6° relative to peaks arising from other crystallographic planes implies that nearly all the sample was converted to crystalline GIC, although a small volume fraction of unintercalated graphite remains [Figure 1 a  (i) and b (i)]. Alkali‐metal GICs are stable only under inert conditions;^13^ upon exposure to air the higher order stage 1 reflections disappear, indicating a decrease in crystallinity of the stage 1 GIC. However, even after extensive washing, the (002) and (003) reflections remain, albeit with reduced intensity, confirming that some residual Na‐THF remains in the sample [Figure 1 b (ii)]. The phase B structure, in which sodium is coordinated to two THF molecules in a “lying down” configuration with an interlayer spacing of 7.2 Å, has been reported when phase A is exposed to air (Figure S2, Supporting Information).^13^ However, no peaks related to the phase B structure were observed. The appearance of a further signal at 2*θ*=25.3° is assigned to the presence of a so‐called “random stage” structure,^43^ where the stacking of hexagonal carbon layers and Na‐THF layers is so disordered that all (00*l*) diffraction lines, except the line due to the most probable spacing of 3.52 Å, are broadened to the extent of being undetectable. The random stage structure may be defined as areas with strongly disordered stacking, with different numbers of carbon layers separating each layer of intercalant (Figure S3, Supporting Information). Alternatively, the observed peak at 2*θ*=25.3° can be attributed to turbostratic graphite,^47^ formed by exfoliation in solution, followed by imperfect restacking on drying.

The XRD patterns for Na‐DMAc‐NFG indicate a new intercalation compound (Figure 1 c), with slightly different interlayer spacings to Na‐THF‐NFG, suggesting that DMAc coordinates to the sodium cations. Peaks at 2*θ*=12.5° and 25.0° correspond to an interlayer spacing of 7.1 Å, similar to the stage 1B structure in Na‐THF‐NFG (Figure S2, Supporting Information). The diffraction peaks were indexed as (00*l*) lines by using 7.1 Å as the identity period. Only the lower reflections are present, suggesting a lower crystallinity in Na‐DMAc‐NFG compared with Na‐THF‐NFG under the same synthesis conditions. With a similar intercalated layer thickness of 3.75 Å (compared to 3.9 Å for Na‐THF‐NFG), it is assumed that the DMAc molecules also adopt a “lying down” arrangement around each sodium cation (Figure S2, Supporting Information). As with the Na‐THF‐NFG sample, the peak at 2*θ*=25.5°, showing no higher or lower stage reflections and corresponding to a layer thickness of 3.5 Å, is assigned as areas of randomly‐staged or turbostratically stacked graphite; a decisive assignment is prevented by the breadth of the feature. Whilst the Na‐DMAc‐NFG stage 1 compound shows less long‐range order than Na‐THF‐NFG, no clearly defined peak at 2*θ*=26.6° was observed, suggesting that more effective intercalation or exfoliation of the layers has been achieved in DMAc, although with a lower degree of crystallinity in the resulting GIC. After the sample was quenched in air and washed, the random stage phase and S1(002) peaks reduced in intensity relative to the S1(001) signal, consistent with the loss of some intercalant [Figure 1 c (ii)].

**Figure 2:**
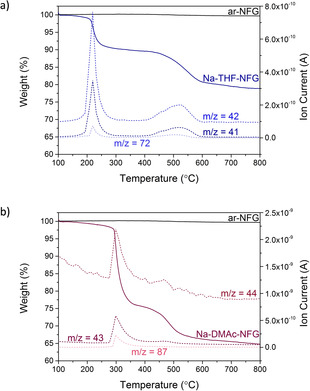
TGA‐MS under N_2_ of a) Na‐THF‐NFG and b) Na‐DMAc‐NFG; corresponding solvent fragments: THF *m*/*z* 41 (‐CHCH_2_CH_2_‐^+^), 42 (‐CH_2_CH_2_CH_2_‐^+^), 72 (C_4_H_8_O^+^); DMAc *m*/*z* 43 (CH_3_CO‐^+^), 44 ((CH_3_)_2_ N‐^+^), 87 (CH_3_CON(CH_3_)_2_^+^). TGA profile of ar‐NFG was provided as a baseline.

TGA‐MS analysis also confirms the presence of residual solvent in the quenched and washed materials (Figure 2). Na‐THF‐NFG shows two distinct weight losses at around 150–250 °C (5.6 wt %) and 400–600 °C (14.6 wt %) (Figure 2 a), both corresponding to the appearance of THF‐related mass fragments *m*/*z* 42 (‐C_3_H_6_^+^), 43 (‐CHCH_2_O^+^) and 72 (C_4_H_8_O^+^), confirming that a significant amount of THF is present in the sample. The absence of mass fragments for *m*/*z=*128, attributed to C_10_H_8_^+^, ionised naphthalene, confirms that naphthalene was fully removed during the washing procedure (Figure S4, Supporting Information). Na‐DMAc‐NFG shows a sharper and higher temperature first weight loss step than Na‐THF‐NFG, between 270 and 380 °C (22.7 wt %), possibly due to DMAc's higher boiling point (165 °C) (Figure 2 b), although the preference of amide solvents for graphene surfaces may play a role.^48^ The second weight loss at 420–550 °C (7.8 wt %) is in a similar temperature range to Na‐THF‐NFG, suggesting that solvent escape at this temperature is not dependent on solvent type. Accompanying mass fragments *m*/*z* 43 (CH_3_CO‐^+^) and 87 (CH_3_CON(CH_3_)_2_^+^) confirm that DMAc is lost during both pyrolysis steps. Both samples show remarkably high stability to temperature, with total solvent removal only achieved above 600 °C. The presence of two well‐defined weight loss steps at different temperatures may indicate the presence of two distinct solvent environments; alternatively, partial solvent decomposition in the first step may produce a less volatile guest that undergoes further degradation at higher temperatures. The loss of DMAc at higher temperatures than THF indicates a better stability of the intercalated solvent. Overall, Na‐DMAc‐NFG contains more residual solvent than Na‐THF‐NFG from the first weight loss step; however, TGA measurements under N_2_ alone were insufficient to conclude whether the solvent is complexed with the Na‐ions or remains as uncoordinated free solvent molecules.

XPS measurements in UHV (ultra‐high vacuum) indicate the sodium content in the ternary Na‐GIC before and after quenching and hence help to quantify the Na/solvent ratios for both systems (Figure S7, Supporting Information). The proportion of THF was estimated from the oxygen content of the GICs after subtracting the oxygen content of ar‐NFG, as a baseline. Before quenching, the XPS‐derived value indicates THF/Na=1.3, consistent with the stage 1B structure, given the approximations of the measurement. However, the oxygen content is difficult to assess accurately by XPS, given the background. Therefore, THF content was also estimated from the TGA mass losses up to 520 °C relative to the subsequent weight loss due to graphitic carbon combustion. The resulting value of THF/Na=3.9 may be a more reliable indicator of stage 1A.

After quenching and work up, most of the sodium is washed away (from 3.93 at % down to 0.6 at %), but the THF/Na ratios are preserved (estimated 5.5 and 3.1 from XPS and TGA measurements, respectively; Table S2, Supporting Information). The results suggest that all residual THF exists coordinated to sodium, and that any free uncoordinated THF is lost during initial drying. Based on TGA, the ratio C/Na increases from 14.3 to 71.2 after quenching (calculated from results obtained from TGA under air, see explanation of Figure S5 and Table S2, Supporting Information) showing that around 80 % of the original sodium was removed by the work up procedure. The sodium content detected by XPS followed a similar trend to the values obtained by TGA, within error.

In the case of Na‐DMAc‐GIC, before quenching, the Na 1s XPS signal can be quantified relative to the N 1s peak uniquely associated with the solvent, to indicate a ratio of DMAc/Na of 1.7. The value correlates with that obtained from the TGA of the quenched sample (2.3). Coordination of DMAc to sodium has not been widely discussed in the literature, and the coordination number and structure is unknown; however, the XPS number suggests a coordination pattern according to the phase B structure (see Supporting Information Table S2 for a summary and further discussion of the TGA and XPS data). Most of the sodium was removed after the quenching and washing procedures (from 2.7 at % down to 0.5 at %). A C/Na ratio of 27.5 (TGA) for Na‐DMAc system compared to 71.2 (TGA) for Na‐THF system after quenching indicates that sodium‐DMAc was much harder to remove than sodium‐THF.

Overall, the data are most consistent with the formation of stage 1 ternary intercalation compounds; for Na‐THF‐NFG a solvent coordination number of 4 is most likely in line with previous reports.^13^, ^20^ For Na‐DMAc‐NFG, calculations based on TGA and XPS suggest a coordination number of 2–3, with the XRD data implying that DMAc molecules coordinate to sodium in a “lying down” configuration (Figure S2, Supporting Information). Most likely, two DMAc molecules coordinate each sodium. In order to understand the evolution of the structure during heating, relevant to both TGA analysis and general expansion/deintercalation, in situ XRD was performed.

In situ XRD was first used to investigate the thermal deintercalation of the Na‐solvent‐NFGs (using Co_Kα1_ rather than Cu_Kα_ radiation, for practical reasons). The XRD patterns for an ar‐NFG control show a small degree of thermal expansion with increasing temperature, ending with an interlayer spacing of 3.39 Å at 700 °C, increased from 3.34 Å (Figure S8 A, Supporting Information). The persistence of the (002) peak at 700 °C shows that ar‐NFG does not decompose in air (Figure S8 B, Supporting Information).

**Figure 3:**
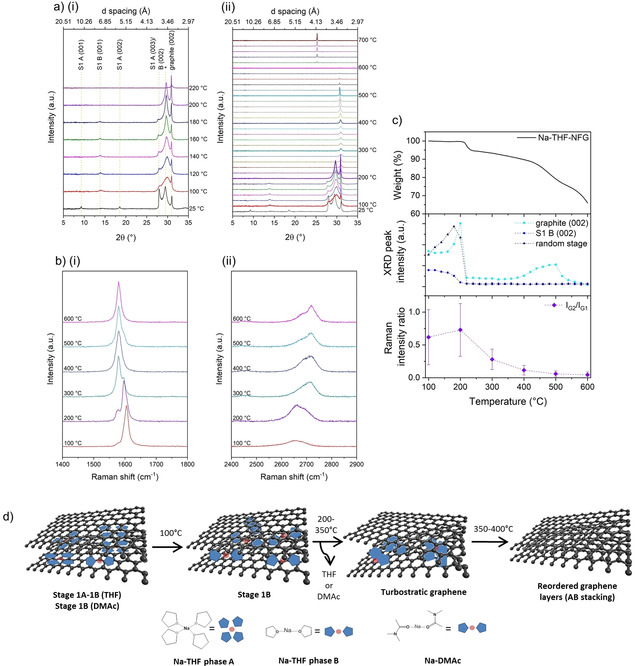
a) XRD patterns of Na‐THF‐NFG at 25 °C, then from 100–700 °C in 20 °C intervals (i) and magnified diffractograms between 25–220 °C (ii); Co_Kα1_ 1.789 Å. Stage 1 phase A and B structures correspond to interlayer spacings of 11.1 and 7.1 Å, respectively. The starred peak is attributed to the “random stage” phase or turbostratic graphite; b) Evolution of Raman G band (i) and 2D band (ii) of Na‐THF‐NFG at 100 °C intervals; c) XRD peak intensity of graphite (002), S1B(002) and random stage structure (middle) and Raman *I*_G2_/*I*_G1_ ratio (bottom) against temperature, with TGA (top) shown for comparison. d) Schematic representation of solvent rearrangement within the graphene layers.

Under the same heat treatment, Na‐THF‐NFG gave rise to dramatically different behaviour (Figure 3). With Co_Kα1_, the characteristic stage 1A peaks visible at 25 °C appear at 2*θ*=9.3° (001), 18.5° (002) and 28.0° (003), whilst the random stage structure peak appears at 2*θ*=29.5° and the graphite (002) peak at 2*θ*=31.1°. The broad peak at 2*θ*=8.1° is assigned to the silica glass sample holder (Figure S10, Supporting Information). At 100 °C, a conversion of stage 1A to 1B was observed from the complete loss of the A(001) and A(002) peaks; the loss of the A(003) reflection is masked by the appearance of the (002) peak for phase B at the same value of 2*θ*, accompanied by the B(001) peak at 2*θ*=14.1° (*d*=1.71 Å) (Figure 3 a). The random stage peak shifted to a higher angle (2*θ*=29.9°) reflecting the loss of solvent intercalant, and broadened indicating even greater heterogeneity within the sample, consistent with the idea that solvent must travel along the interlayer galleries to the edge of the graphene sheets before it is lost (Figure 3 d). Given that phase B is known to form upon exposure of phase A to ambient conditions over a long time period, it is not surprising that this restructuring was observed during the early stages of heating.^13^ As the temperature increased to 200 °C, the remaining stage 1 phase B Na‐THF converted to the random stage or turbostratic phase, evidenced by the decreasing 2*θ* position of B(002) due to layer expansion during solvent loss, a steady decrease in peak intensity and its eventual total loss by 200 °C (Figure 3 a). No defined higher stage compounds appear [see Table S1 for *d* (001) values, Supporting Information], so the structure must evolve through a disorganised mixture of different stages, represented by the random stage peak, which sharpens and intensifies, also with a slight downshift in 2*θ*. Concurrently, the graphite (002) peak becomes stronger, suggesting the recovery of some graphitic domains, presumably from the “closing up” of layers as intercalant pockets coalesce. A similar phenomenon can be observed by TEM when the solvent pockets are irradiated by the electron beam, where moving pockets visibly expand and travel towards sheet edges (Figure S9, Supporting Information). Between 200 and 220 °C, the XRD behaviour changes dramatically: all well‐defined GIC stage structuring is lost from the sample, coinciding with the first weight loss step in the TGA showing the removal of THF; as the solvent evaporates, crimping the edge of the graphene layers and trapping the solvent gas as “bubbles”, the interlayer spacing expands, with only the crimped region contributing to the broadened graphite peak as “bubbles”. At the same time, this peak loses significant intensity, suggesting that remaining ordered areas of graphite were expanded during this solvent loss, with graphene layers being forced apart to allow THF molecules to escape.

Upon further heating, the sample showed a slow recovery of graphitic domains, with assumed sliding/coalescing of uncoordinated solvent pockets between the layers towards flake edges following the Daumas–Herold model,^47^ with an acceleration around 400 °C; this temperature coincides with the second weight loss step in the TGA. As the THF molecules reach the critical temperature and escape from disordered pockets, gradually the graphene layers reorder, regaining graphitic AB stacking. After 520 °C, the graphite (002) peak decreases and then disappears, as the expansion associated with further solvent escape accelerates the onset of combustion. Eventually all carbon material disappeared, leaving a white solid on the disc with a distinct peak at 2*θ*=25.3°, most likely due to devitrification of the silica sample holder to form a small fraction of cristobalite.^49^

The trend seen by XRD is corroborated by Raman spectra of samples taken in the same temperature range (Figure 3 b). Between room temperature and 300 °C, splitting of the G band is observed (Figure S11 in the Supporting Information and Figure 3 b), a common phenomenon in materials with incomplete doping^48^ where the signal at a lower wavenumber (≈1580 cm^−1^) arises from one graphene layer surrounded only by other graphene layers (G1), as in undoped graphite. The band with higher shift (≈1605 cm^−1^) is attributed to a graphene layer which is adjacent to an intercalant layer (G2). The ratio *I*_G2_/*I*_G1_ therefore indicates the degree of intercalation. At 200 °C and below, *I*_G2_ */I*_G1_ is around 0.7 (Figure 3 c), implying a significant amount of doping in the sample, but the very broad distribution indicates that the sample is inhomogeneous with pockets of pristine graphite as well as fully intercalated stage 1 areas. Above 200 °C, however, *I*_G2_/*I*_G1_ decreases and the distribution narrows suggesting a recovery of graphitic stacking after solvent escape, supporting the results obtained from XRD. Changes in the 2D signal also reflect changes in the structure of Na‐THF‐NFG upon heating (Figure 3 b). At 100 °C, the signal is downshifted (2650 cm^−1^) and symmetrical, typical of intercalated or exfoliated materials; as the sample is heated, the 2D band shifts (2700 cm^−1^) and the characteristic shoulder is restored, indicating recovery of graphitic stacking.

**Figure 4:**
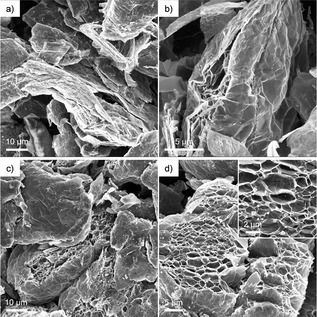
SEM images of Na‐THF‐NFG after heating under nitrogen to a,b) 300 °C, and c,d) 650 °C. A much greater expansion can be seen at the higher temperature, consistent with XRD.

Overall, a striking correlation can be seen between the XRD, Raman and TGA data during heating (Figure 3 c). In the temperature range where solvent loss is detected by TGA (200 °C), there is a concomitant decrease in the (002) peak, signalling the expansion of graphene layers. Leading up to these temperatures where solvent is lost, there is a modest increase in (002) intensity, suggesting that intercalant rearrangement occurs with increasing temperature (150 °C), where the driving force is the restacking of graphitic layers. As the temperature reaches a critical solvent desorption temperature, depending on the strength of chemisorption (≈200 °C for THF), there is a rapid escape of gaseous solvent molecules and an expansion of the graphite compared to the starting material. Proof this behaviour is visible by SEM (Figure 4 a,b and Figure S12, Supporting Information). After a certain amount of solvent escapes, the pressure is alleviated, and the edges may partially close up again, preventing further solvent loss (200–380 °C). As the sample is heated further, the remaining solvent continues to coalesce (500 °C), allowing further restacking of graphene layers, reflected in the decrease of Raman *I*_G2_/*I*_G1_, and the return of a more graphitic nature observed in the 2D peak. However, the weak and broad (002) peak in XRD implies that these graphitic domains have very little long‐range order. Once a critical pressure is reached, possibly associated with solvent decomposition, the THF escapes completely, irreversibly expanding the overall graphite structure; SEM images show these open pockets (Figure 4 c,d), where it can be seen that the structure has expanded even further.

**Figure 5:**
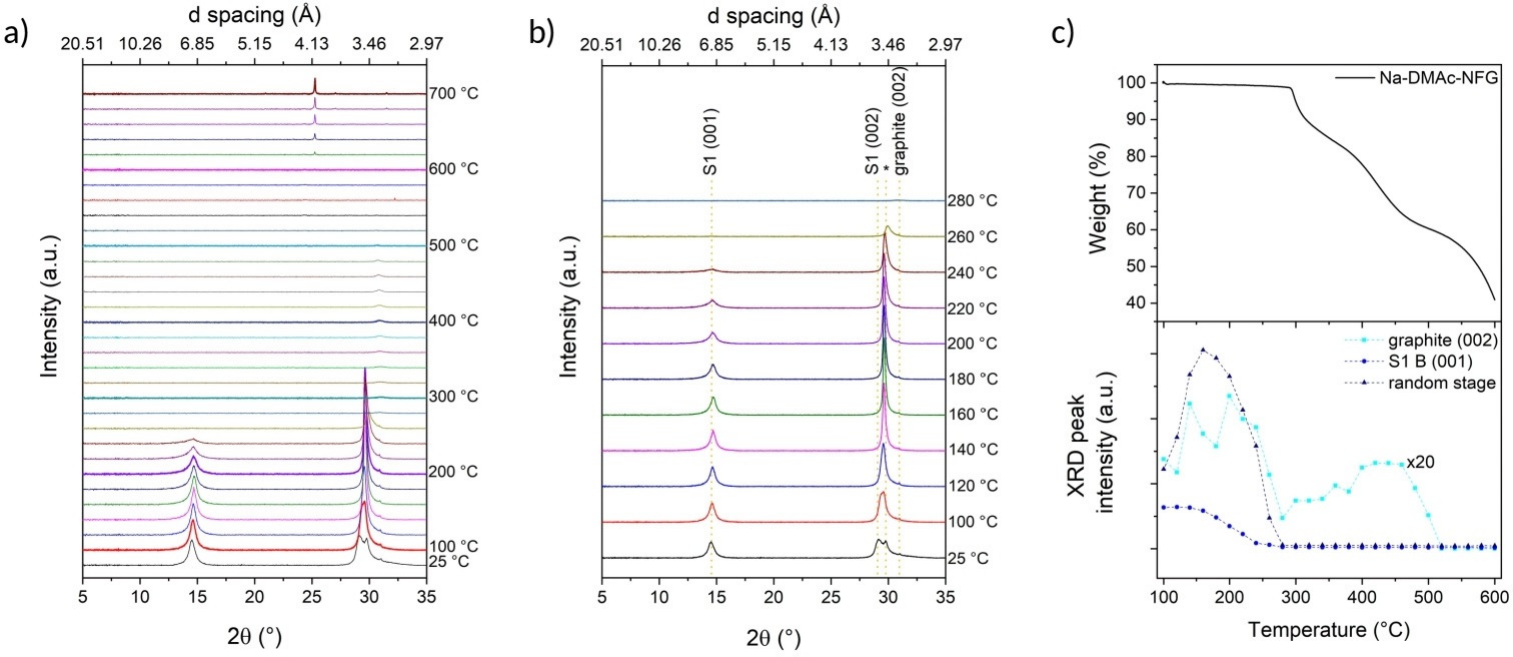
a) XRD patterns of Na‐DMAc‐NFG at 25 °C, then from 100–700 °C in 20 °C intervals, and b) magnified diffractograms between 25–280 °C; Co_Kα1_ 1.789 Å. Stage 1 structure corresponds to an interlayer spacing of 7.1 Å; the starred peak is attributed to the “random stage” phase or turbostratic graphite; c) XRD peak intensity of graphite (002), S1B(001) and random stage structure against temperature, with TGA (under N_2_) shown for comparison.

Na‐DMAc‐NFG shows a related behaviour; initially, at 25 °C, the intercalate *d* layer peaks appear at 14.5° (*d*=1.75 Å) and 29.1° (*d*=3.41 Å), the random stage phase at 29.8°, overlapping with the S1(002) peak, and a very weak graphite (002) peak at 31.0° (Co_Kα1_ radiation, Figure 5). Between 25 and 100 °C, both stage 1 peaks sharpen slightly and shift to higher 2*θ*, suggesting that the interlayer spacing contracts while increasing the degree of order (Figure 5 b). The random stage phase peak downshifts, gradually merging with the S1(002) signal; above 100 °C, this combined signal sharpens and intensifies, while the graphite (002) peak becomes stronger and the S1(001) peak weakens and broadens, suggesting that the stage 1 compound gradually disappears and converts into the random stage phase by 260 °C. These observations are consistent with the graphene layers closing up as uncoordinated solvent pockets coalesce and travel towards flake edges. The onset of the first weight loss is reflected by the sudden decrease in intensity of the random stage signal, and by 280 °C, all trace of defined GIC structure is lost and the graphite peak weakens and broadens, coinciding with the first solvent loss step, where DMAc molecules escape from flake edges. A gradual increase in the graphite (002) intensity follows up to the second loss of DMAc from the sample; as seen before in Na‐THF‐NFG, the solvent molecules coalesce within the interlayer galleries, allowing a “closing up” of graphene layers and therefore a recovery of the (002) peak. In the second weight loss, the sample is fully expanded as solvent bursts from the material, and thereafter, the peak signal further decreases due to combustion of the sample, leaving an inorganic residue, again, most likely due to conversion of amorphous SiO_2_ to cristobalite.^49^ The trend in the graphite (002) peak intensity with temperature is similar to in Na‐THF‐NFG (Figure 5 c), but much less defined, since the original (002) peak signal was extremely weak, appearing over a broad background, resulting in much less reliable curve fitting data.

Overall, these XRD measurements during heating indicate that the Na‐solvent‐NFGs are surprisingly stable in ambient conditions at room temperature. Gradual rearrangement of the staging structure occurs up until the first expansion step (generally around 200–300 °C), where some solvent is lost from the sample, and all distinct staging is lost. Interestingly, rearrangement during these temperatures does not proceed via other well‐defined higher stage intercalation compounds, since no peaks were detected for these structures. Following the first solvent loss step, there is a gradual annealing of the remaining graphite, reflected in the increase in the graphite (002) peak. This rearrangement proceeds until the second loss of residual solvent, from large solvent bubbles that likely burst leaving the large openings observed in SEM (Figure 4) and causing further exfoliation, without any long‐range order in XRD. From the TGA and XRD data, it appears that the temperature at which the first weight loss occurs is dependent on both the strength of the solvent coordination to the sodium and its boiling point, whereas the second step is independent of the solvent type. TGA is the most commonly used technique for determining grafting ratios of functionalised 2D nanomaterials; understanding the solvent trapping behaviour should improve accuracy, as the presence of solvent is frequently not taken into account. TGA coupled with other techniques such as GC or MS, along with suitable calculations, will allow more reliable grafting ratios to be obtained.^38^, ^50^

## Conclusion

Ternary sodium GICs were prepared using THF and DMAc as the co‐intercalant, and the formation of a stage 1 intercalation structure was confirmed by XRD, TGA‐MS and XPS analysis. This first report on the intercalation of Na‐DMAc into graphite indicates an interlayer spacing of 7.1 Å, consistent with a “lying down” configuration of the DMAc molecules. Surprisingly, both compounds are kinetically stable even after exposure to air and remain so at elevated temperatures. Thermal deintercalation of both Na‐THF‐NFG and Na‐DMAc‐NFG results in the loss of solvent, but without rearrangement to any well‐defined higher stage compound. At high temperature, when any ordered GIC structure is disrupted, graphite pockets still trap residual solvent. This deintercalation behaviour is different to electrochemical deintercalation of KC_8_, where the graphite completely recovers the original graphitic structure.^51^ These results have implications for the quenching and work up procedure following reductive grafting of graphites, as opposed to other nanocarbons with curved morphologies (such as CNTs). Caution is needed when estimating the GRM grafting ratios, since the amount of residual intercalant will depend on the flake size, initial degree of exfoliation and layer number of the starting graphite; natural flake graphite sheets might be expected to trap a larger degree of solvent due to their large size and this solvent trapping effect is likely to be mitigated with the use of alternative graphite starting materials.

## Experimental Section

### Materials

Natural flake graphite (NFG) was obtained from Graphexel Ltd. (grade: 2369) and used without any further purification. Tetrahydrofuran, dried in‐house in a solvent‐drying tower packed with alumina, was degassed via a freeze‐pump‐thaw method then further dried over 20 vol% 4 Å activated molecular sieves. *N*,*N*‐dimethylacetamide (Sigma–Aldrich, anhydrous grade) was dried over 20 vol% 4 Å activated molecular sieves. Naphthalene (99 %, Sigma–Aldrich) was dried under vacuum in the presence of phosphorus pentoxide before use. Sodium (99.95 %, ingot) was purchased from Sigma–Aldrich and used as received.

### Experimental procedures

**Preparation of sodium‐naphthalide solution**: A stock sodium‐naphthalide solution was prepared to allow for accurate, simple addition of sodium to dried natural flake graphite (ar‐NFG). 23 mg (1 mmol) sodium and 128 mg (1 mmol) dried naphthalene were added to 10 mL degassed anhydrous THF or DMAc in a N_2_‐filled glove box, and stirred for 1 day until all sodium had dissolved, forming a dark‐green solution.

**Synthesis of Na‐solvent‐NFG**: A Young's tube containing graphite (15 mg, 1.25 mmol carbon) and a magnetic stirrer bar was heated at 400 °C for 1 h under vacuum, and then kept under vacuum for 16 h at room temperature, before placing in a glove box. 1.04 mL of either sodium‐naphthalide solution was added to the Young's tube and the concentration of graphite in THF or DMAc adjusted to 0.1 m by addition of 11.46 mL of the corresponding solvent (C/Na=12, [Na]=0.008 m). The suspension was stirred at room temperature for 2 days under N_2_. For inert characterisation, the mixture was filtered through a 0.1 μm PTFE membrane (Millipore), and washed thoroughly with THF or DMAc/THF before mounting directly onto the sample holder and covering with Kapton tape for XRD measurements. This same sample was used for inert TGA and XPS measurements. To quench the product, dry O_2_/N_2_ (20/80 %, ≈1 L) was bubbled into the solution for 15 min, then stirred overnight under dry O_2_/N_2_ to quench any remaining charges. The mixture was filtered through a 0.1 μm PTFE membrane and washed thoroughly with THF, ethanol and water to remove any residual naphthalene and sodium salts formed during the reaction. The product was obtained as a dark grey powder after washing with ethanol and drying overnight under vacuum at 80 °C.

### Equipment and characterisation

Thermogravimetric analysis coupled with mass spectrometry (TGA‐MS) was performed using a Mettler Toledo TGA/DSC 1 instrument integrated with a Hiden HPR‐20 QIC EGA mass spectrometer under nitrogen atmosphere. Samples were held at 100 °C for 30 min, then heated from 100 to 850 °C at 10 °C min^−1^ (N_2_ flow rate=60 mL min^−1^). X‐ray photoelectron spectroscopy (XPS) data were recorded using a K‐alpha^+^ XPS spectrometer equipped with an MXR3 Al_Kα_ monochromated X‐ray source (h*ν*=1486.6 eV). X‐ray gun power was set to 72 W (6 mA and 12 kV). Charge compensation was achieved with the FG03 flood gun using a combination of low energy electrons and the ion flood source. Survey scans were acquired using 200 eV pass energy, 1 eV step size and 100 ms (50 ms×2 scans) dwell times. All high‐resolution spectra were acquired using 20 eV pass energy, 0.1 eV step size and 1 s (50 ms×20 scans) dwell times. Samples were prepared by pressing the sample onto carbon‐based double‐sided tape. Pressure during measurement acquisition was ≤1×10^−8^ mbar. Atomic compositions were calculated from averaged spectra obtained from at least 3 areas per sample. Raman spectra were collected on a Renishaw inVia micro‐Raman (1000–3000 cm^−1^), using a 50 mW 532 nm laser at 10 % laser power. Statistical Raman data were obtained from measurements carried out in Streamline mode of at least 500 areas per sample. Samples were prepared by drop casting dispersions on a glass slide or silicon wafer. UV/Vis‐NIR absorption spectra were measured with a PerkinElmer Lambda 950 UV/Vis spectrophotometer, using a quartz cuvette with 1 cm pathlength. Transmission electron microscopy (TEM) was carried out using a JEOL 2100Plus TEM at 200 kV operating voltage. Samples were prepared on 300 copper mesh holey carbon grids (Agar Scientific) by drop‐casting dilute graphene dispersions onto a grid supported by filter paper and drying under vacuum. Ambient XRD data was recorded on a PANalytical X′Pert PRO diffractometer operating at 40 kV and 40 mA, with Cu_Kα_ (*λ*=1.542 Å) radiation, at a scan rate of 0.085° s^−1^, step size of 0.0334°, and 2*θ* varying between 5° and 60°. Dried powder samples (5–10 mg) were mounted onto a zero‐background Si sample holder (PANalytical Ltd., UK) and levelled to the height of the top of the holder using a glass slide. Non‐ambient XRD measurements were acquired on a PANalytical X′Pert PRO MPD diffractometer equipped with monochromated cobalt radiation (Co_Kα1_, *λ*=1.789 Å), operating at 40 kV and 30 mA. The diffractometer was fitted with an Anton Paar HTK 1200 n sample stage allowing operation from room temperature to 1200 °C. The samples were heated in air to 700 °C and X‐ray diffraction patterns (Co_Kα1_) were collected at 20 °C intervals from 100 °C. The sample was prepared by spreading a thick slurry of the graphite material in ethanol onto a thin silica glass disk, ensuring a uniform flat surface after evaporation of the solvent. The silica disk was then secured onto the alumina sample carrier and mounted within the heating chamber. The heating and data acquisition programs were controlled using X′pert Data Collector software. Heating of the sample was conducted in an air environment. The temperature was increased at 10 °C min^−1^ between measurements and held isothermally during data acquisition. Measurements were taken at 25 °C, and thereafter from 100 to 700 °C in 20 °C intervals. Scans were taken from 5–40° with a step size of 0.0167° and a scan rate of 0.0167° s^−1^. SEM images were taken using a Leo Gemini 1525 field emission gun scanning electron microscope (FEGSEM) with SmartSEM software, at an accelerating voltage of 5 keV, working distance of about 7 mm and a 30 μm aperture. Powder samples were fixed onto aluminium stubs using carbon tabs (Agar Scientific Ltd.).

## Conflict of interest

The authors declare no conflict of interest.

## Supporting information

As a service to our authors and readers, this journal provides supporting information supplied by the authors. Such materials are peer reviewed and may be re‐organized for online delivery, but are not copy‐edited or typeset. Technical support issues arising from supporting information (other than missing files) should be addressed to the authors.

Supplementary
