# Supplementary material for: Thermal Decomposition of Ternary Sodium Graphite Intercalation Compounds
Source: Chemistry. 2020 Apr 7;26(29):6545–53. doi: 10.1002/chem.202000422 (PMC7317426; doi:10.1002/chem.202000422)
Supplement: Supplementary file 1 — Supplementary [file CHEM-26-6545-s001.pdf]

Chemistry–A European Journal

Supporting Information

# Thermal Decomposition of Ternary Sodium Graphite Intercalation Compounds

Heather Au+,[a, b] Noelia Rubio+,[b] David J. Buckley,[c] Cecilia Mattevi,[d] and Milo S. P. Shaffer*[b]

# SUPPLEMENTARY INFORMATION

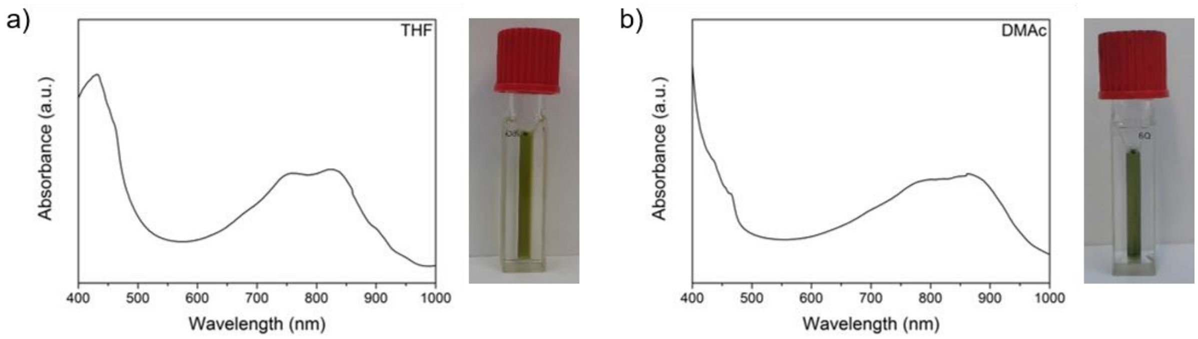


*Figure S1. UV-vis spectra and digital photographs of sodium naphthalide in a) THF and b) DMAc. Each solvent system shows the characteristic naphthalide radical anion peak at ~800 nm.*

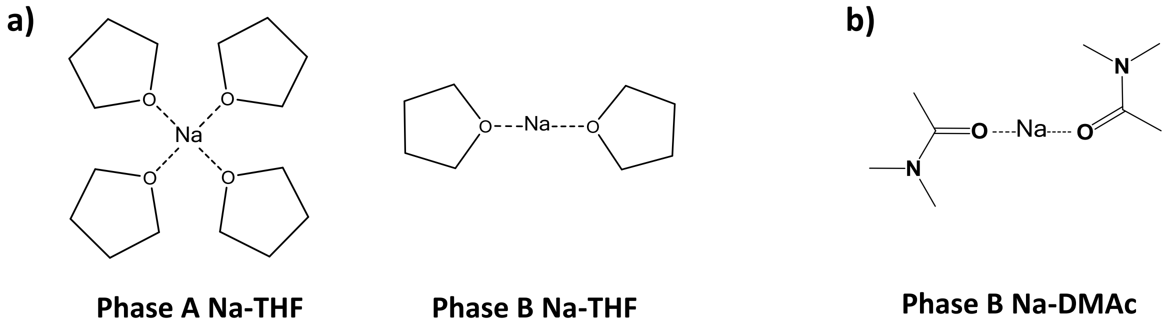


*Figure S2. a) Phase A and B structures of Na-THF. b) Hypothesised Na-DMAc coordination.*

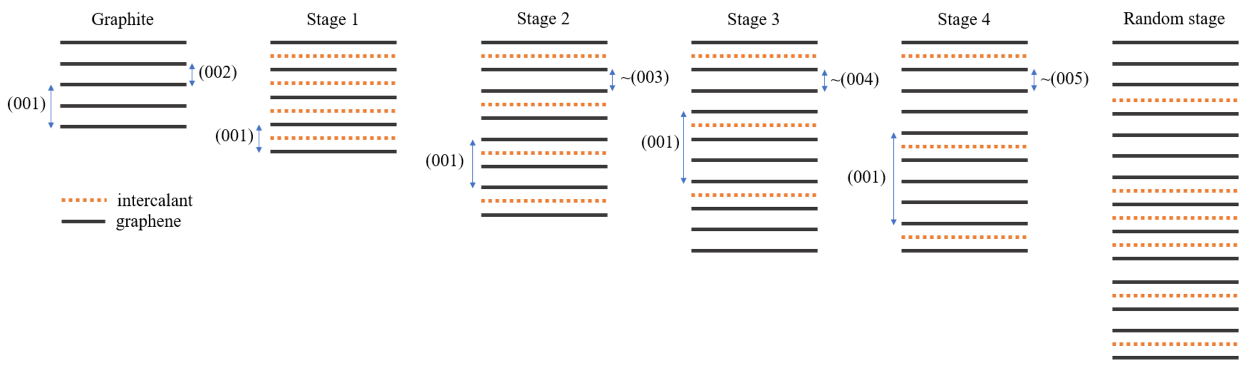


*Figure S3. Illustration of the different stages found in graphite intercalation compounds. In the random stage structure, diffraction lines for each indexed structure (arising from the regular repetition of carbon and intercalate layers) are so much broadened (due to the small crystallite size) that they are not detected, except for the signal at 25.3°, which may be considered an overlap of many different 00l reflections from different stage compounds.*

*Table S1. Calculated d(001) values for Na-THF-GIC, based on intercalate layer thickness of 7.8 Å for a phase A compound and 3.9 Å for phase B,[1] and their corresponding CoK$\alpha_1$ shifts.*

| | d(001) phase A (Å) | 2θ (°) | d(001) phase b (Å) | 2θ (°) |
|---|---|---|---|---|
| **Stage 1** | 11.2 | 9.16 | 7.2 | 14.3 |
| **Stage 2** | 14.6 | 7.0 | 10.6 | 9.7 |
| **Stage 3** | 17.9 | 5.7 | 14.1 | 7.3 |
| **Stage 4** | 21.3 | 4.8 | 17.5 | 5.9 |

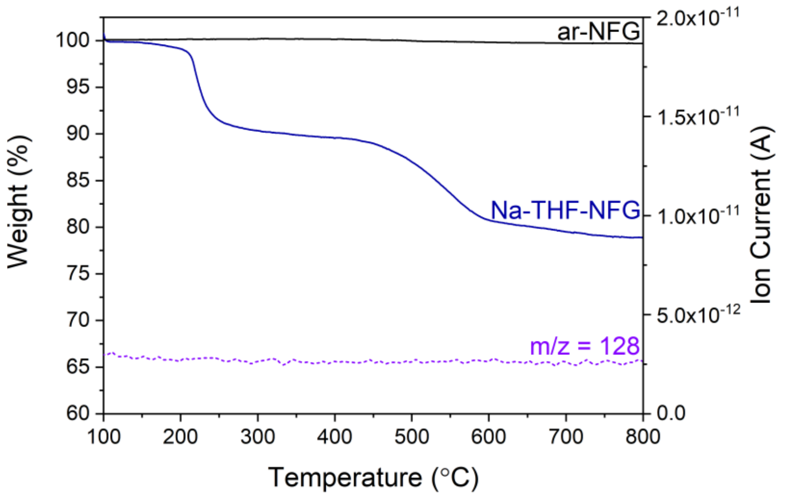


*Figure S4. TGA-MS profiles under $N_2$ of Na-THF-NFG and ar-NFG, and absence of naphthalene ion m/z 128 ($C_{10}H_8^+$).*

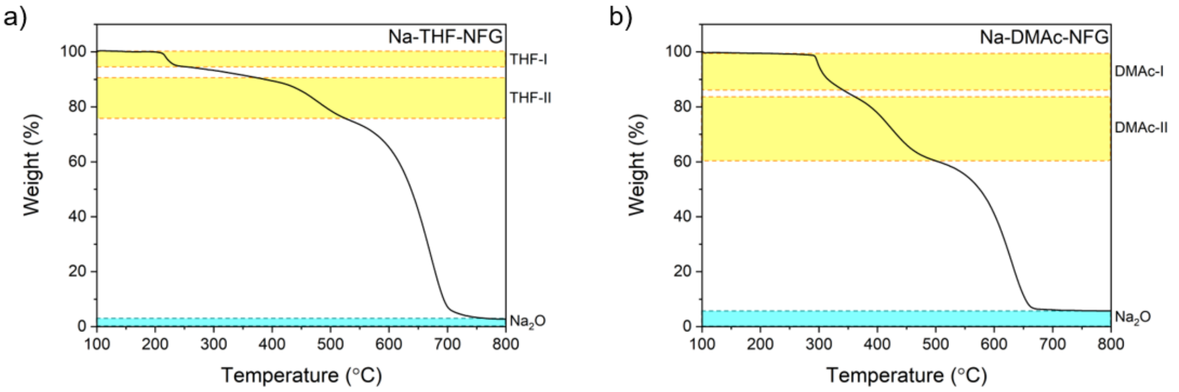


*Figure S5. TGA under air of Na-solvent-NFGs to estimate sodium composition.*

An estimate of the amount of residual sodium inside the sample was obtained by TGA in air. After combustion, a white solid remained in Na-THF-NFG, so assuming all sodium converted to sodium oxide after 800 °C, and graphite left no remaining char, the residual mass gives a sodium oxide content of 2.8 wt% of the total sample and therefore a sodium content of 2.0 wt%. Taking the total amount of THF from the mass losses around 170-260 °C (THF-I) and 370-520 °C (THF-II) (Table S1), and attributing the remaining weight loss to combustion of graphitic carbon, a ratio of THF/Na = 3.1 was obtained, suggesting that THF exists coordinated to sodium in a mixture of phase A and B regions, and that any free uncoordinated solvent is likely lost during initial drying. A C/Na = 71.2 indicates that most of the original sodium was removed with around one sixth remaining after the work up procedure. The same calculations for Na-DMAc-NFG result in a DMAc/Na ratio of 2.3, and a C/Na of 27.5.

TGA of sealed GIC-NFG under air revealed the amount of solvent trapped in between the layers before quenching and work up. Special aluminium pans were used for this purpose. Samples were loaded into the pans inside the glovebox; the pans were then sealed with a hand press and transferred to the TGA instrument. This instrument had the facility to puncture the lid immediately before the experiment (experiments are run under nitrogen atmosphere). However, the maximum operating temperature for aluminium pans is 500°C; thus the amount of sodium was determined in a further experiment using normal alumina pans heated up to 850°C under air atmosphere (Figure S5 and Table S2).

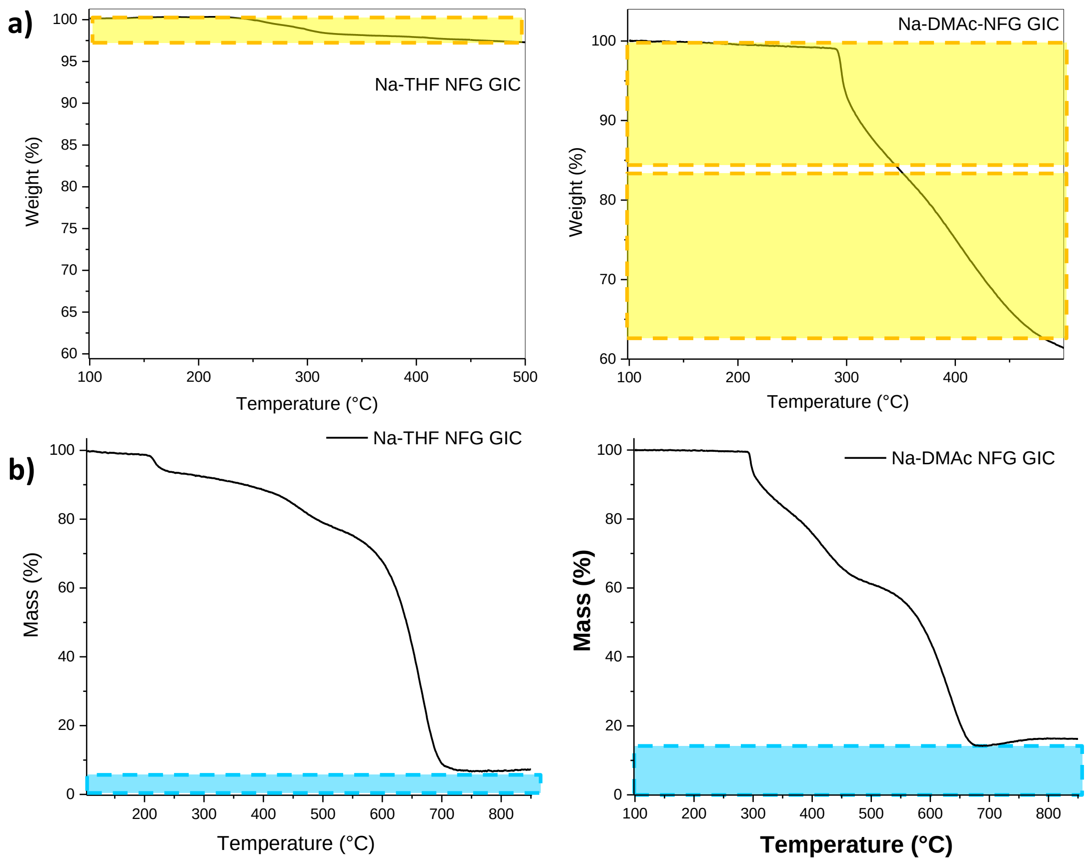


*Figure S6. (a) TGA under air of NFG-GIC using sealed aluminium pans. (b) TGA under air of NFG-GIC to determine the sodium residue.*

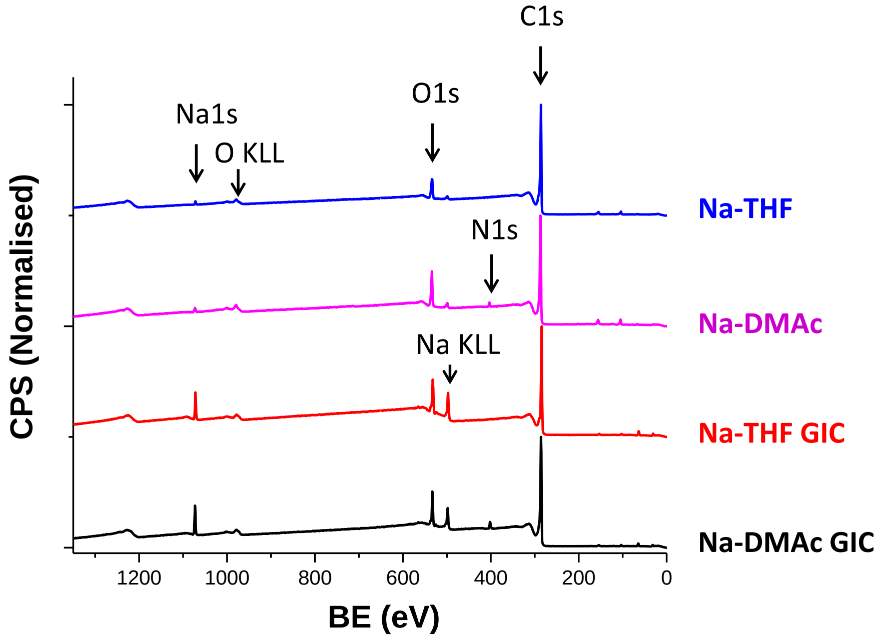


*Figure S7. XPS measurements in ultra-high vacuum of the different graphite systems.*

*Table S2. Summary of solvent content in ar-NFG and Na-solvent-NFG, by TGA and XPS. TGAs were carried out under air atmosphere.*

| | TGA | | | | | XPS | | | | | |
|---|---|---|---|---|---|---|---|---|---|---|---|
| **Sample** | solv-I (wt%)* | solv-II (wt%)* | $Na_2O$ (wt%)* | solv/ Na* | C/Na * | at% C† | at% O† | at% Na† | at% N† | solv/ Na† | C/Na † |
| **ar-NFG** | - | - | - | - | | 95.0 | 5.0 | 0 | - | - | |
| **Na-THF-NFG** | 5.6 | 14.6 | 2.8 | 3.1 | 71.2 | 88.9 | 10.5 | 0.6 | - | 5.5 | 111.5 |
| **Na-DMAc -NFG** | 14.0 | 22.6 | 5.7 | 2.3 | 27.5 | 86.2 | 11.6 | 0.5 | 1.7 | 3.4 | 158 .8 |
| **Na-THF-GIC-NFG** | 1.8 | 5.1 | 7.0 | 3.9 | 14.3 | 85.8 | 10.3 | 3.93 | - | 1.3 | 16.9 |
| **Na-DMAc GIC-NFG** | 15.9 | 22.6 | 14.3 | 3.6 | 4.8 | 85.8 | 9.15 | 2.7 | 2.4 | 1.7 | 28.2 |

*Calculated from TGA measurements under air atmosphere; †obtained from XPS.

GIC-NFG samples for XPS measurements were prepared inside the glovebox to avoid air exposure. The samples were deposited as normal on the XPS sample holder inside the glovebox and then a special enclosure was used to transfer the samples from the glovebox into the XPS instrument loadlock chamber. This setup allows the transfer of the samples under vacuum (~$10^{-2}$ mbar); once the setup is exposed to XPS ultra high vacuum atmosphere (~$10^{-7}$ mbar) the holder automatically opens, allowing the XPS experiments to be performed without air exposure.

XPS quantification of the THF content relies on the oxygen component; however, there is significant intrinsic oxygen content (~ 4-5 at %) in the as-received material, which may also vary during reduction, creating significant uncertainty. On the other hand, for Na-DMAc samples, XPS is more reliable as it can exploit the unique signal from the nitrogen atom in the solvent.

Conversely, the TGA samples are easier to prepare for the more volatile THF-containing GICs. After filtration in the glovebox, the reactive GIC samples were transferred directly into the sealed aluminium pans for TGA measurement under nitrogen, whereas samples for XPS were dried under high vacuum before measurement. Since DMAc is less volatile than THF, it is likely that far more residual uncoordinated solvent remained in the Na-DMAc-GIC sample prepared for TGA, overestimating the solvent:Na ratio by this route. It was possible to more thoroughly wash and dry the quenched samples (Na-THF-NFG and Na-DMAc-NFG) after removal from the glove box. The quenched samples, in principle, should give similar ratios, but with lower accuracy due to the lower absolute Na content. In particular, the XPS is likely to be less reliable

due to the removal of the intercalated species from the near the surfaces to which XPS is sensitive.

Overall, therefore, arguably the most reliable measurements for the solvent:Na ratio are given by the XPS of the GIC for the DMAc, and the TGA of GIC for the THF. Both Na-DMAc-GIC-NFG (XPS) and Na-DMAc-NFG (TGA) indicate a solvent:Na ratio around 2. Both Na-THF-GIC-NFG and Na-THF-NFG indicate a solvent:Na ratio 3-4 by TGA.

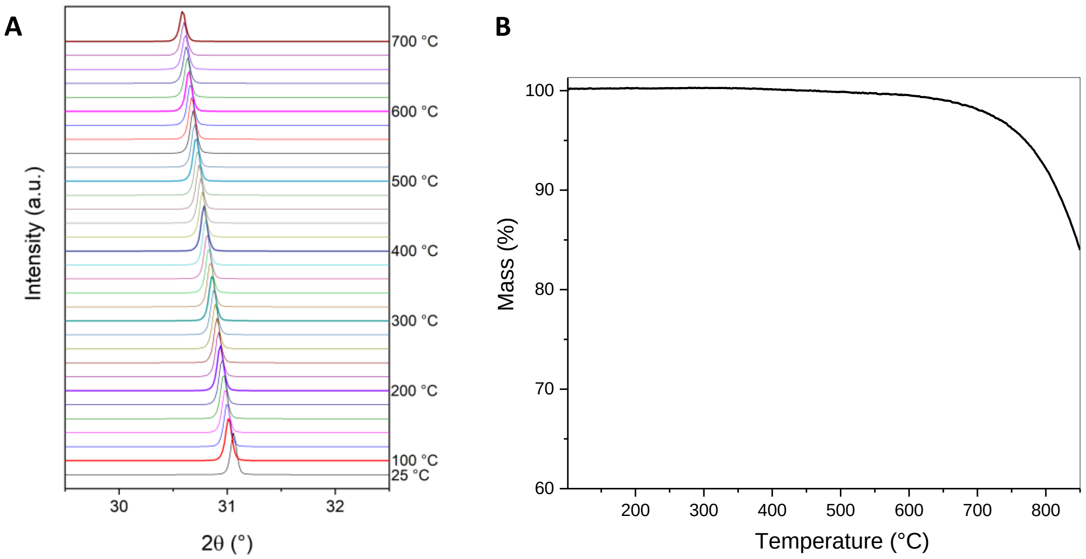


*Figure S8. A) XRD patterns of ar-NFG, at 25 °C, then from 100-700 C in 20 °C intervals; CoK$\alpha_1$ 1.789 Å. B) TGA under air of ar-NFG.*

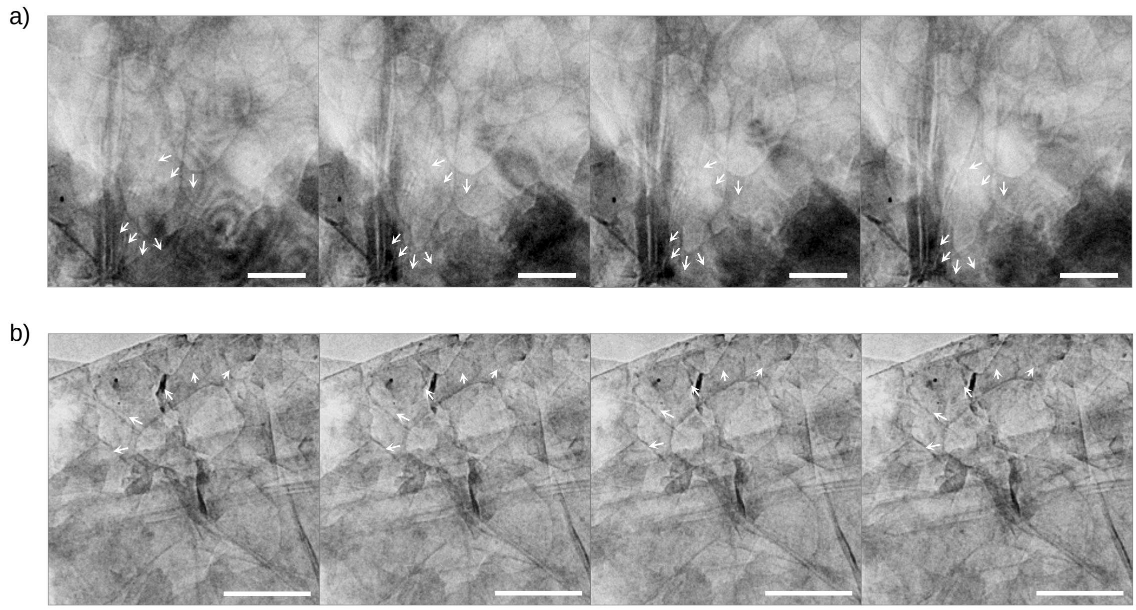


*Figure S9. Time-lapse TEM images of pocket areas growing and travelling under the electron beam, in two different regions shown in a) and b) for Na-THF-NFG; scale bar is 100 nm.*

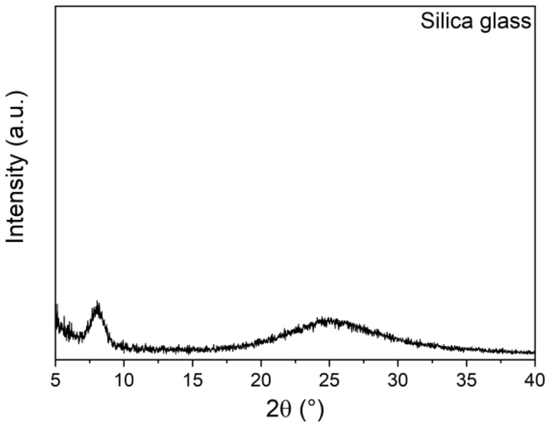


*Figure S10. XRD of blank silica glass showing a peak at 8.1° and a broad feature around 20-30°.*

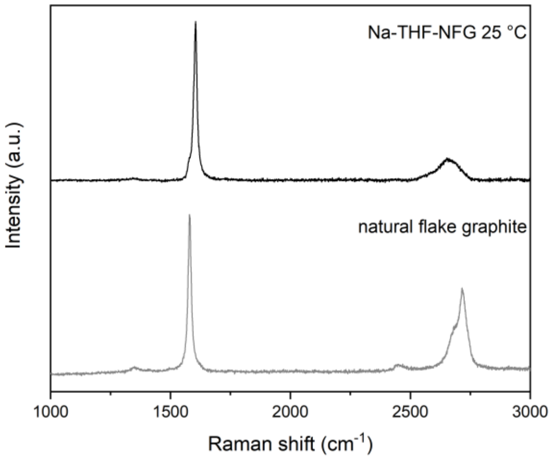


*Figure S11. Raman spectra of a) pristine graphite and b) Na-THF-NFG.*

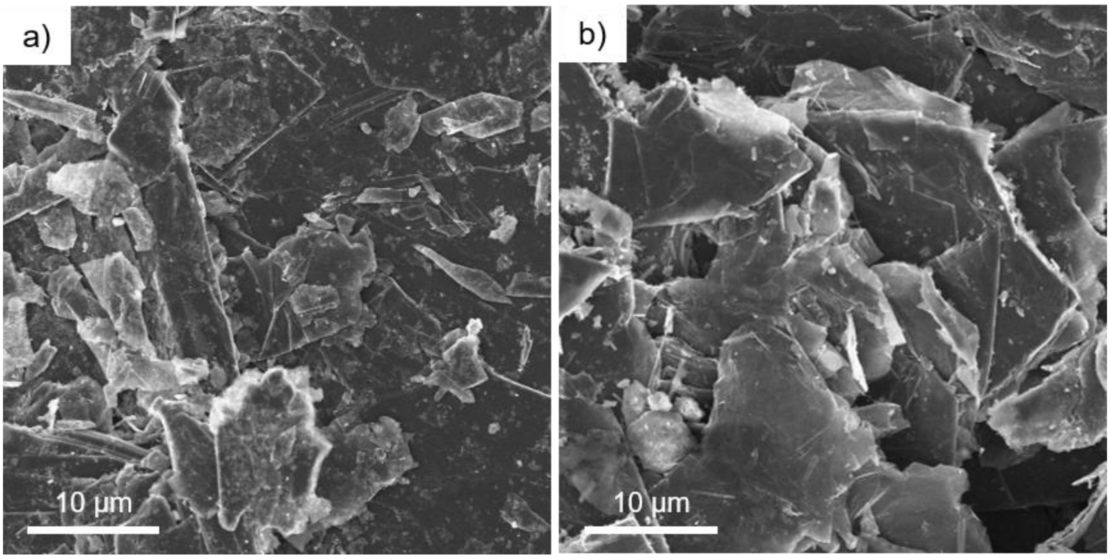


*Figure S12. SEM images of a) as-received natural flake graphite, and b) Na-THF*

## References

1. M. Inagaki and O. Tanaike, *Synthetic Metals*, 1995, **73**, 77-81.
